# A missense mutation in *LIM2* causes autosomal recessive congenital cataract

**Published:** 2008-06-23

**Authors:** Surya Prakash G. Ponnam, Kekunnaya Ramesha, Sushma Tejwani, Jyoti Matalia, Chitra Kannabiran

**Affiliations:** 1 Kallam Anji Reddy Molecular Genetics Laboratory, Hyderabad Eye Research Foundation, LV Prasad Eye Institute, Hyderabad, India; 2 Jasti V Ramanamma Children’s Eye Care Centre, L V Prasad Eye Institute, Hyderabad, India

## Abstract

**Purpose:**

To identify mutations in the *LIM2* gene in families with hereditary congenital or juvenile-onset cataract.

**Methods:**

Forty families (total of 100 affected and 84 unaffected individuals) were recruited for the study. Probands were screened for pathogenic alterations in 10 different candidate genes including the lens intrinsic membrane protein-2 (*LIM2*) gene. Exons and flanking regions were screened by polymerase chain reaction (PCR) amplification, single-strand conformation polymorphism, and sequencing. Sequence changes were evaluated in 75 unrelated normal controls.

**Results:**

A missense mutation, Gly154Glu, was found in *LIM2* in one family with four individuals diagnosed with autosomal recessive cataract from two generations. An evaluation of seven individuals (four affected and three unaffected) showed that the mutation was homozygous in the affected members and heterozygous in unaffected members tested. It was absent in 75 unrelated ethnically matched normal controls. All affected individuals had a severe phenotype of congenital cataracts and visual impairment.

**Conclusions:**

The Gly154Glu mutation involves a non-conservative change that presumably results in loss of function of the MP19 protein. This study shows the involvement of *LIM2* in human congenital cataract.

## Introduction

Blindness due to cataract represents a major cause of treatable blindness in different parts of the world [1-3]. Cataract in infants and children carries the risk of irreversible visual loss or amblyopia due to improper visual inputs to the brain, and cataract-related amblyopia accounts for 8% of blindness in children in southern India [4]. Loss of transparency of the lens can be a result of multiple factors that cause changes in the cellular or macromolecular organization in the lens, which result in light scattering and opacification. Hereditary cataracts arising from single gene mutations have provided insights into some of the structural and functional requirements for lens transparency. So far, 16 genes are identified to cause autosomal dominant congenital cataract [5], and eight genes are identified for autosomal recessive cataract [5-8].

The lens intrinsic membrane protein-2 gene (*LIM2*) encodes an abundant integral lens membrane protein of 19 kDa, MP19 (alternatively known as MP17/MP18/MP20). The function of MP19 is not clearly understood as yet. It localizes to junctional regions of the lens fiber cell membrane as well as throughout fiber cell membranes, suggesting a role in lens junctional communication [9,10]. MP19 has been shown to be absent from proliferating epithelial cells in the lens with expression becoming prominent in differentiating cells as well as in mature lens fiber cells [11,12]. It binds calmodulin [13,14] as well as galectin, a protein associated with lens cell membranes [15]. While various models have been proposed for MP19 topology, it is predicted to have four transmembrane segments and two extracellular [16,17] with NH_2_- and COOH-termini in the cytoplasm or with the NH_2_-terminus integrated into the membrane [18].

Evidence for the role of *LIM2* in cataract came initially from the *To3* mouse, an ENU (ethyl nitroso-urea)-induced mutant that demonstrated a semi-dominant cataract that mapped to the same region as *Lim2* on mouse chromosome 7 [19]. While both heterozygous and homozygous mutants had dense cataracts, homozygotes also had microphthalmia, disorganization of lens fibers, and rupture of the lens capsule [19,20]. The *To3* mutation was identified as a Gly15Val change in *Lim2* [20]. A similar cataract phenotype was also reproduced in transgenic mice carrying the *Lim2* transgene with the same mutation [21]. *Lim2* homozygous knockout mice were found to have pulverulent nuclear opacities and altered refractive properties of the lens whereas heterozygotes had normal lenses [22], suggesting that loss of function of *Lim2* is responsible for the phenotype. To date, there has been one report of human hereditary cataract arising from a mutation in *LIM2* in a family with autosomal recessive presenile cortical cataract with a missense mutation, Phe105Val [23].

Here, we report a homozygous missense mutation in *LIM2* in a family with autosomal recessive cataract of congenital onset.

## Methods

### Patients and sample collection

Probands presenting at the pediatric ophthalmology clinic of our institution with a diagnosis of congenital or developmental cataract were recruited for the study based on an ophthalmic evaluation by slit lamp biomicroscopy of probands and available family members by two independent ophthalmologists. Inclusion criteria were the presence of a bilateral cataract of congenital or developmental type based on history and/or examination. Exclusion criteria were a history of trauma, unilateral (non-familial) cataract, co-existing ocular disease, mental retardation, microcephaly, cerebral palsy, systemic syndromes, and maternal history of intrauterine infections or antenatal steroid use. After prior approval of the protocol by the Institutional Review Board of the L.V. Prasad Eye Institute (Hyderabad, India), informed consent was obtained from participants,  blood samples (2-8 ml) from the probands and family members were collected in heparin-coated vacutainers by venipuncture, and family history, pedigree, and clinical data were recorded. Forty families were recruited as part of the study. They consisted of 30 families with dominant cataracts and 10 families with recessive cataracts with a total of 100 affected and 84 unaffected individuals.

### Mutational analysis

Genomic DNA was extracted from blood leukocytes by the standard phenol-chloroform method. Ten candidate genes (six crystallin genes, two connexin genes, *LIM2*, and the *heat shock factor 4* (*HSF4*) gene) were screened for mutations in probands from all families. The gene sequences were retrieved from the Ensembl database. Suitable primers for polymerase chain reaction (PCR) amplification of exons and flanking sequences of *LIM2* were designed using primer design software. Primer sequences used are as shown in Table1. PCR was performed by using 50 ng of DNA template, 5 pmol each of forward and reverse primers, 1 unit of Taq DNA polymerase (Bangalore Genei, Bangalore, India), and 0.2 mM dNTPs in a total volume of 25 μl. Cycling conditions were as follows: initial denaturation at 94 °C for 2 min and 35 cycles of denaturation at 94 °C for 30 s, annealing at 51–53 °C for 30 s, and elongation at 72 °C for 45 s followed by one cycle of final extension at 72 °C for 8 min.

**Table 1:** Details of polymerase chain reaction primers for *LIM2* amplification.

**Exon**	**Primer name**	**Primer sequence (5′-3′)**	**Product size (bp)**	**Annealing Temp (°C)**
I	LIM 1F	CCATTGTGTAGGGAGGCTTA	213	52
	LIM 1R	AGGTCCTGGGAGAAGAAGG		

II	LIM 2AF	CAGTTCCTCCCTTCAAGTCC	159	53
	LIM 2AR	ACTGCATCCAGTGGTCTGTT		
	LIM 2BF	TGTACAGCTTCATGGGTGGT	255	52
	LIM 2BR	TGGAATACAGGTGTCCTTGG		
	LIM 2CF	TACCTGCAGACAGACAGCAT	238	52
	LIM 2CR	CCCAACTTAACCTTCAAACC		

III	LIM 3F	TCATCTCAGAGGTAGCAGCA	279	52
	LIM 3R	ATTGGGGTTTGAGATGAGAG		

IV	LIM 4F	AAAATCACACCCAGCCTTAG	248	51
	LIM 4R	ACTCTATCTGCTGCCCACTC		

V	LIM 5F	GGTGTTGGGCTCTCTTG	231	51
	LIM 5R	CTAGGAACCAGGATTTCA		

Sequences of forward (F) and reverse (R) primers used for amplification of *LIM2* exons are shown above along with sizes of PCR products for each primer pair. Exon 2 was amplified in three fragments using primer sets denoted 2A-2C.

PCR products were mixed with two volumes of formamide. Samples were denatured by heating at 95 °C for 5 min and then snap chilled. The products were then subjected to electrophoresis on 8% polyacrylamide gels containing 5% glycerol. All samples were electrophoresed at room temperature and at 4 °C. The variants observed on single-strand conformation polymorphism (SSCP) analysis were identified by bi-directional dideoxy sequencing of the relevant PCR products using fluorescent automated methods. Screening for the observed mutation or variation was performed on DNA from 75 ethnically matched, unrelated normal controls using SSCP. Family members of the proband were tested to check for cosegregation of the sequence change with disease. Designation of sequence changes is according to the cDNA sequence of human *LIM2*. Human *LIM2* sequences were obtained from the Vega genome browser; human transcript ID OTTHUMT00000151603 and gene ID OTTHUMG00000071186. The mouse *Lim2* genomic sequence (gene ID ENSMUSG000000046134) was from Ensembl.

To assess the probable effect of an amino acid substitution on the protein, SIFT (Sorting Intolerant from Tolerant) scores were obtained by using SIFT software. SIFT aligns sequences homologous to the protein of interest from the databases and predicts whether a specific amino acid substitution will be tolerated by calculating normalized probabilities (range from 0 to 1) for each substitution at a particular position. Scores below a threshold of 0.05 are predicted to be deleterious and those above the threshold are predicted as tolerated [24,25].

## Results

**Figure 1:**
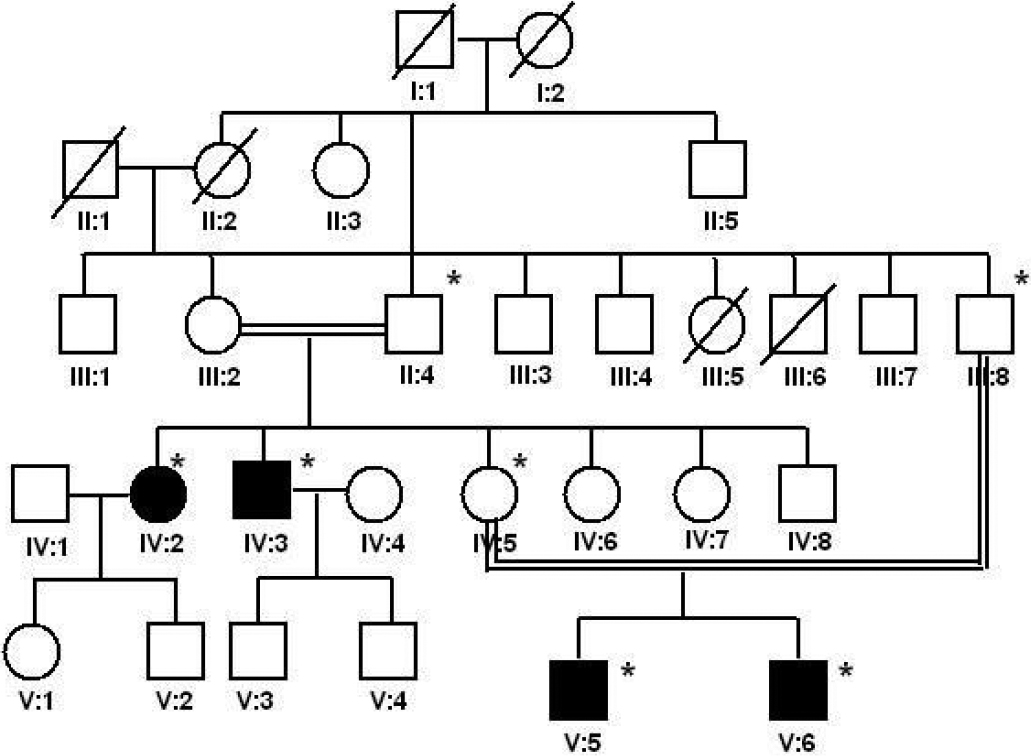
Pedigree of family with autosomal recessive cataract. Dark circles and squares represent affected females and males, respectively, and open symbols represent unaffected individuals. Symbols with an asterisk show individuals on whom genetic analysis was done.

Screening of probands from 40 families for pathogenic mutations revealed one mutation in *LIM2* in one family (pedigree in Figure 1). There were four affected individuals from two generations (IV:2, IV:3, V:5, and V:6 in Figure 1). All four individuals were offspring of consanguineous marriages. In addition to the four affected members, three other unaffected relatives-the mother (IV:5), the father (III:8), and the maternal grandfather (II:4) of the proband-were evaluated.

**Figure 2:**
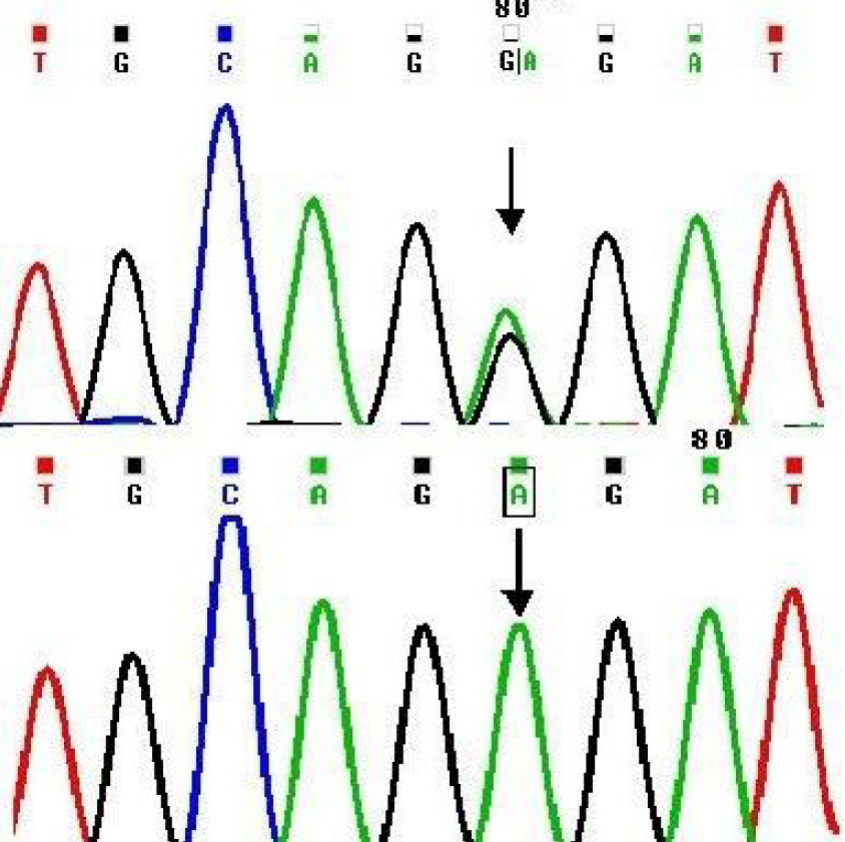
Sequence electropherogram showing mutation in *LIM2*. Heterozygous c.587G>A change (arrow) found in unaffected members of the family (see Figure 1) involving GGG>GAG (Gly154Glu) codon change is shown in the top panel. The bottom panel shows homozygous G>A at this position in affected individuals (arrow at site of mutation).

Sequence analysis showed a change of c.587G>A in human *LIM2* cDNA (Figure 2) involving a codon change of GGG>GAG, which leads to a predicted glycine-154 to glutamic acid substitution (Gly154Glu). All four affected individuals in the family were homozygous for the mutant allele (Figure 2, bottom), and the three unaffected individuals tested were heterozygous (Figure 2, top). The change was absent in 75 ethnically matched normal controls.

The clinical features of the members analyzed are presented in Table 2. The proband presented to us at six years of age and was pseudophakic in both eyes with dense amblyopia. He had reportedly been affected since the age of three years, at which time he had cataract surgery. His visual acuity was 6/60 (20/200) in the right eye and counting fingers at 1 m in the left eye. His affected sibling was reported to have cataract since he was two years old. He was four years old at presentation and when he had cataract surgery. The proband’s maternal aunt and uncle also had congenital and developmental cataract respectively and were operated for cataract removal within the first decade of their lives (Table 2). All affected individuals had nystagmus and dense amblyopia.

**Table 2:** Mutational and clinical status of individuals from family with the *LIM2* mutation.

**Individual**	**Relation with proband**	**Age at presentation**	**Age at onset**	**Genotype**	**Clinical status**
V:5	Proband	6 years	3 years	GGG>GAG (homozygous)	Total cataract, surgery at 3 years, amblyopia
V:6	Sibling	4 years	1.5 years	GGG>GAG (homozygous)	Total cataract, surgery at 4 years, poor fixation, amblyopia
IV:2	Maternal aunt	35 years	Birth	GGG>GAG (homozygous)	Surgery at 3 years, nystagmus, amblyopia
IV:3	Maternal uncle	29 years	<8 years	GGG>GAG (homozygous)	Surgery at 10 years, nystagmus, amblyopia
III:8	Father	35 years	-	GGG>GAG (heterozygous)	Normal
IV:5	Mother	27 years	-	GGG>GAG (heterozygous)	Normal
II:4	Maternal grandfather	55 years	-	GGG>GAG (heterozygous)	Normal

Pedigree of the family is shown in Figure 1. The numbers for each individual in the left column are according to the pedigree drawing.

## Discussion

The mutation described here is the second known human mutation in *LIM2* causing hereditary cataract. It differs from the Phe105Val mutation reported by Pras and coworkers [23] in that the associated phenotype reported in their study was relatively mild with a late-onset of cataract, pulverulent cortical opacities, and mild or moderate visual loss. All affected individuals in the family studied by us had congenital cataracts evident at or shortly after birth with severe visual impairment as indicated by the presence of nystagmus and amblyopia. The absence of a phenotype in heterozygous carriers points to a loss of function of the Gly154Glu mutant MP19 protein as a possible cause of the disease. A substantial impact of the Gly154Glu mutation on protein structure is suggested by the non-conservative nature of the substitution, involving the replacement of a neutral, polar small amino acid (glycine) by a charged, larger amino acid (glutamic acid). Further, introduction of a charged amino acid into this position, which is predicted to be located within the fourth transmembrane segment of MP19 [16], would be presumably incompatible with the structure and topology of the protein in its wild type form. It is possible that the mutant may not be targeted to the membrane thus resulting in loss of function.

We used the SIFT tool [25] to assess the probable impact of the Gly154Glu substitution as compared with the Phe105Val found in human cataract [23] and the Gly15Val in *To3* mouse [20]. This action yielded a score of 0 for the Gly154Glu mutation, thereby interpreting it as deleterious to protein function. A deleterious effect is also predicted for the mouse Gly15Val mutation, which gave a score of 0.03. On the other hand, the Phe105Val substitution has a SIFT score of 0.24, predicting that it would be tolerated. The predicted low impact of the Phe105Val mutation on the protein is compatible with the mild cataract observed in the individuals carrying the mutation [23]. However, the dominant effect of the *To3* (Gly15Val) mutant is not explainable from existing data. A dominant negative effect of the *To3* mutation was also suggested in studies on the *Lim2 ^To3^* transgenic mice, which demonstrated severe cataracts despite the amount of transgenic *Lim2* mRNA present being much lower than wild type *Lim2* mRNA [21]. Chen and coworkers [26] have shown that the MP19 *To3* protein accumulates within the cytoplasm of transfected cells while the wild type MP19 localizes to the plasma membrane. They proposed that MP19 *To3* is a deleterious gain of function mutant that might be cytotoxic in lens fibers. In contrast, homozygous *Lim2* knockout mice had relatively mild changes consisting of pulverulent central opacities and changes in refractive properties of the lens [22]. Comparison of these latter observations with the recessive mutation identified in our study may imply a different role for MP19 in mice and humans. The effect of these mutations on the MP19 protein cannot be gauged fully until its function and physiological effects are understood.

The location of the Gly154 codon spans two exons including bases at the 3′ boundary of exon 4 and 5′ boundary of exon 5. The second base of the codon, which is the site of the G>A mutation, is located at the 5′ end of exon 5 next to the splice acceptor site. This raises the possibility that this mutation might have an additional effect on mRNA splicing, which needs to be investigated further.

The present study provides further evidence for the importance of *LIM2* in maintaining the normal structure and function of the lens and demonstrates for the first time that a mutation in *LIM2* results in human autosomal recessive congenital cataract.
